# Direct and Indirect Pathways of Variation in Length of Exposure to the Majority Language, Cognitive and Language Skills in Preschoolers’ Listening Narrative Comprehension

**DOI:** 10.3390/children8080636

**Published:** 2021-07-26

**Authors:** Raffaele Dicataldo, Maja Roch

**Affiliations:** Department of Development and Socialization Psychology, University of Padova, 35131 Padova, Italy; raffaele.dicataldo@unipd.it

**Keywords:** listening narrative comprehension, language exposure, inferential abilities, theory of mind, preschoolers

## Abstract

Listening narrative comprehension, according to the theoretical framework of the multicomponent model for comprehension, involves numerous skills that interact dynamically between each other and have the potential to give rise to individual differences in comprehension. The purpose of the current work was to define a comprehensive and complete multicomponent model of listening narrative comprehension in preschool age. We investigated how variation in Length of Exposure to majority Language (i.e., how long children have been exposed to the Italian language), lower-order cognitive (WM, inhibitory control, attention shifting), language skills (receptive vocabulary, syntactic knowledge, rapid naming), and higher-order cognitive skills (inferences, TOM, knowledge of story-structure) are related to listening narrative comprehension in Italian of 111 preschool children (M_age_ = 61 months; SD = 6.8) growing in a monolingual or multilingual context. Structural equation modeling results showed that the model explained 60% variance in listening narrative comprehension in Italian of children aged four to six and predicted the outcome both through direct and mediated paths, coherently with the multicomponent model of comprehension.

## 1. Introduction

Narratives surround children from their earliest language experiences [1,2,3]. Young children experience narratives through shared book reading and participation in talks about daily events. Children as young as two to three years old develop a rich repertoire of knowledge about narratives and use that to narrate their needs, desires, plans, to understand, and respond to others’ demands, requests, needs, and emotional reactions [4]. Beyond these rudimentary experiences with narrative stories, young children become increasingly skilled at understanding and producing narratives [5].

Considering the pervasiveness of narratives in children’s life, the assessment of narrative skills from the early stages of language development becomes crucial. Measures of narrative skills, both in production and comprehension, provide, in fact, many and rich information concerning the linguistic development of children in an ecologically valid way and thus represent an intriguing research area for understanding the relationship between different levels of linguistic processing [6]. Storytelling or retelling of pictured stories are the tasks mostly used to assess narrative ability. These tasks allow detecting lexical knowledge and a level of acquisition of morphosyntactic structures (micro-structure level) and global coherence and cohesion (macrostructural level) [7]. Storytelling and retelling are also considered valuable clinical tools by providing valuable information on children’s linguistic and metalinguistic knowledge [8,9,10,11].

In this study, we focused on the comprehension of a listened narrative. Listening narrative comprehension is defined as the process by which lexical information, sentences, and other information are interpreted [12], allowing the building of a coherent mental representation of the meaning of a narrative [13]. Listening narrative comprehension is usually measured through a comprehension questions task which follows a short narrative read by an adult while the child is listening. Questions used to assess comprehension are focused on both the information presented in the narrative and on information that has to be inferred based on the events narrated in the narrative [14,15]. The child is required either to provide a verbal answer or to choose an alternative among fillers [15,16]. Assessing young children’s narrative comprehension through listening tasks allows to minimize the constraints of oral language skills involved in narrative production tasks and to minimize interpretation difficulties that children might encounter when interpreting pictured stories.

In this study, the theoretical framework of the multicomponent model for comprehension [1] was adopted. According to this model (described below), language and cognitive skills involved in narrative comprehension interact dynamically and reciprocally allowing to reach a coherent and mental representation of the narrative’s meaning [1]. The majority of studies that adopted a multicomponent approach focused on reading comprehension in school-aged children [17,18], whereas systematic investigation on narrative listening comprehension and its components in young children has not received much attention until recently [14,19]. The importance of listening narrative comprehension from preschool age was recognized since listening narrative comprehension during preschool age resulted to be the best predictor of later reading comprehension [20,21,22]. These few studies on listening narrative comprehension in preschoolers tended to focus on few linguistic and cognitive skills. Results provide piecemeal evidence and scarce information about structural relations among language and cognitive skills involved [23]. We still have much to understand about how language and cognitive skills influence each other, and how they become integrated to produce successful narrative comprehension [3].

Other important aspects that rarely have been considered in listening narrative comprehension are the individual differences in children’s language and cognitive skills, strictly related to environmental factors. Individual differences in children’s language and cognitive skills are related to early life experiences that can have a profound impact on the developing brain and its organization [24,25]. Development and early experience change the brain’s physical structure and functional organization, allowing it to adapt to its environment [26].

Exposure to and experience with one or more languages have been found to impact children’s developmental trajectories, yielding variation in their linguistic and cognitive profiles [27]. It is well known that time and opportunity to hear and use a language influence the development of language and thus children’s performance in oral language skills and listening narrative comprehension [28,29]. Nowadays, most classes are composed of children who have a variable exposure to the majority language spoken at school, as many children come from multilingual backgrounds. The variation in the exposure to the majority language among children raised in multilingual contexts accounts for individual differences in linguistic and cognitive development [30,31,32] and should be included as a factor in a multicomponent model of listening narrative comprehension.

This work aims to examine, within the multicomponent model of comprehension framework, direct and indirect pathways of a large and comprehensive set of language and cognitive skills involved in listening narrative comprehension. Participants are children, aged between 44 to 75 months, growing in monolingual and multilingual contexts. We examined partial and complete mediations of several language and cognitive skills to listening narrative comprehension. Participants were monolingual and bilingual children exposed to Italian who varied for their length of exposure to that language since we were interested in analyzing whether the variation in the length of exposure to the majority language affects their narrative listening comprehension, over and above the effects of all the linguistic and cognitive components involved.

Relevant literature that guided this study is reported below, divided according to thematic paragraphs that correspond to the goals of the work.

## 2. Multicomponent Model of Listening Narrative Comprehension

As reported above, in this study we adopted the multicomponent model of text comprehension [1], which has been partially tested for narrative listening comprehension in preschoolers [15,16,33]. According to this model, narrative comprehension involves several language and cognitive skills, which are included in one of three broad categories, namely, lower-order cognitive and language skills and higher-order cognitive skills, and entailed in two different levels of processing [34]. The first level of processing (lower level) involves components that allow basic processing of linguistic and cognitive information, such as vocabulary and grammar, memory, and attention. The second level of processing (higher level) involves components that allow reaching a coherent global representation of text meaning, such as inferential abilities, integration of previous knowledge with text information and knowledge of story structure [1].

The different components of listening narrative comprehension develop during preschool age and become more and more efficient. Additionally, their role in understanding oral narratives is not the same: some of the components have a direct role in determining individual differences in narrative comprehension, others are related to narrative comprehension only by playing an indirect role, i.e., mediated by another skill. Following is the literature review of the relevant studies that investigated the direct or mediated role of the various components in narrative comprehension.

### 2.1. The Direct and Mediated Role of Lower-Order Cognitive Skills in Listening Narrative Comprehension

Listening narrative comprehension is a complex ability requiring the integration of several different lower-order cognitive skills. This set of skills comprises skills within the executive functions, namely, that set of cognitive processes that is necessary for the cognitive control of behavior. Executive functions include basic cognitive control components such as attentional control, cognitive inhibition, inhibitory control, working memory, and cognitive flexibility [35].

Since listening narrative comprehension requires remembering words and sentences, holding and retrieving information from previous parts, and relating text information to background knowledge, it would seem obvious the involvement of the working memory in this process. Working memory is the capacity to store and manipulate information see [36] for a review. Previous studies found that working memory (measured by backward task) predicts narrative comprehension in preschoolers better than short-term memory (measured through a forward task) [16,37]. Currently, there is no clear evidence about the nature of the contribution of working memory in listening narrative comprehension. Strasser and del Rio [19] found that inferential abilities and comprehension monitoring partially mediate the effect of WM on listening narrative comprehension. Kim and Phillips [23] found that working memory was indirectly related to listening narrative comprehension via higher-order cognitive skills (comprehension monitoring and ToM). Florit and colleagues [16], on the other hand, found that memory skills, measured through a forward and backward span test, provided a unique and independent contribution to listening narrative comprehension after controlling for verbal skills, explaining 10% of the variability in listening narrative comprehension in children aged between four and six years.

Another lower-order cognitive skill that was hypothesized to be important for listening narrative comprehension is the ability to inhibit a strong response in favor of a weaker but more appropriate one [38]. The ability to inhibit the attention to irrelevant details, focusing on central elements may lead children to have more resources available for successful comprehension. Kim and Phillips found that inhibitory control was positively and directly related to listening comprehension after accounting for vocabulary and age, whereas Strasser and del Rio did not find this relation [14,19].

The third lower-order cognitive skill involved in narrative comprehension is the ability to focus attention on relevant stimuli to solve a task and to shift attention from one stimulus to another as needed [39]. Previous studies have shown that sustained attention predicted story generation outcomes [9,40]. It seems reasonable to speculate that it may represent an important cognitive resource also for successful listening narrative comprehension. To the best of our knowledge, the only study that investigated the relationship between sustained attention and listening comprehension is the study conducted by Kim [14]. In her work with first graders, it emerged that sustained attention was related to lower-level linguistic skills, namely, vocabulary and grammar, but was not related to higher-order cognitive skills, and listening comprehension after accounting for the other variables included in the model.

In conclusion, although there are some indications of the contribution of cognitive resources to listening narrative comprehension, it is still unclear what kind of contribution these components make to narrative comprehension, i.e., whether it is directed or mediated by verbal skills and high-level cognitive skills.

### 2.2. The Direct and Mediated Role of Lower-Order Linguistic Skills in Listening Narrative Comprehension

The second group of components described in the multicomponent model of text comprehension includes linguistic skills necessary for the processing of the narrative text, namely, vocabulary and syntax knowledge, and rapid naming.

Vocabulary knowledge represents the core ability and one of the best predictors of narrative comprehension from kindergarten to school in whatever modality a text is presented [16,41,42]. Kendeou and colleagues showed that among the sample of four-year-olds with middle-class backgrounds, receptive vocabulary was one of the two unique predictors, with inferential ability, of narrative listening comprehension [41]. Moreover, Florit and colleagues found that expressive and receptive vocabularies specifically account for later listening comprehension through both direct and indirect effects [43]. It may be argued that both vocabularies uniquely account for listening comprehension [44,45].

Vocabulary itself is not sufficient for a successful comprehension, in fact, also syntactic knowledge is needed [17]. Syntactic knowledge is the knowledge of how words can be combined in meaningful sentences, phrases, or utterances [46]. Few studies have investigated its relation with listening narrative comprehension, but its role is not yet clear. Florit and colleagues found that the role played by sentence comprehension is fully mediated by basic semantic, lexical, and cognitive components, showing that syntactic knowledge necessary for understanding isolated sentences does not play a crucial role in establishing the meaning of a text, at least when word knowledge and verbal working memory are taken into account [15]. To date, only one study investigated mediation via higher-order skills, of syntactic knowledge in listening narrative comprehension [47]. The author found that syntactic knowledge was directly related to narrative listening comprehension as well as indirectly via comprehension monitoring and ToM.

The third lower-order oral language skill relevant for listening narrative comprehension is rapid naming. The speed with which children name digits, letters, or colors is related to many measures related to reading and literacy, including accurate word decoding, oral reading, and comprehension [48]. Parilla, Kirby, and McQuarrie in a longitudinal study of children from first to third grade, found that rapid naming in kindergarten directly predicted text reading comprehension in all three grades later [49]. The mechanisms underlying the link between rapid naming and reading skill remains unclear [50]. Many investigators have suggested that rapid naming measures a child’s mastery of orthographic codes and their associated phonological codes, thus it measures a child’s ability to execute reading-specific skills automatically [51]. An alternative view is that the link between rapid naming and reading skill reflects more general cognitive processes, not those specific to processes required for skilled reading. Kail and Hall [52], for example, proposed that the naming–reading link reflects a global developmental change in processing speed. According to this view, the correlation between rapid naming and reading reflects the fact that both are linked to age-related changes in processing speed. To the best of our knowledge, there are no studies that investigated the relationship between rapid naming, listening narrative comprehension, and its components, before formal reading acquisition.

To summarize, lower-level linguistic skills are a prerequisite for narrative processing. However, the literature suggests that not all linguistic components have a comparable impact on narrative comprehension. Vocabulary seems to be directly connected to listening narrative comprehension but also through higher-level cognitive skills such as comprehension monitoring and Theory of Mind [23]. The role of sentence comprehension in narrative comprehension seems less clear and is much more likely to be mediated by other skills. Finally, the contribution of rapid naming still needs to be defined, since the link between rapid naming and comprehension, in listening and reading, remains unclear. Therefore, it is relevant to investigate in a single model the contribution of each linguistic component and the type of relationship (direct or mediated) it has with listening narrative comprehension.

### 2.3. The Direct and Mediated Role of Higher-Order Cognitive Skills in Listening Narrative Comprehension

Linguistic skills are not sufficient to adequately understand a narrative: several higher-order cognitive skills are necessary to integrate the information between the text and the previous knowledge. These skills include a wide range of general cognitive skills such as inferential skills, Theory of Mind, and knowledge of story structure.

When children listen to a story, to understand adequately, they must be able to draw spontaneously appropriate inferences [53]. Inferential ability refers to the ability to integrate explicit contents with previous knowledge to derive meaning that is not explicitly stated in the text [3]. The ability to generate inferences has been found to contribute to young children’s ability to understand literal as well as inferred meaning, leading to better listening texts comprehension [33,41,54]. There is strong evidence that inferential skills directly predict narrative listening comprehension, after the role of lower-level cognitive and linguistic skills has been controlled for [3,43,55].

Theory of mind (ToM) refers to the ability to understand own and others’ mental states and to predict behavior [56]. The theory of mind has been recently and more often included in the multicomponent models of narrative comprehension. The evidence collected so far suggests that ToM has a direct relation with different measures of narrative comprehension [19,57]. Theory of mind is important to understand characters’ motivations and mental states, and it is necessary for story construction beyond language ability. Additionally, since ToM has a strong linguistic component that tends to covariate with many other language measures, it would seem useful to analyze all these relations in a comprehensive model of comprehension [57].

Finally, the knowledge of story structure has been also included in some studies on listening narrative comprehension. Story structure refers to the organization found in common children’s stories [58]. Story structure elements include the setting and the main character, an initiating event and reaction, solution attempts, the outcome of these attempts, and the ending reaction. The relationships among these elements can also be expressed by story grammar [58]. Knowledge of story structure acts as a schema that supports comprehension of the narrative [59]. To our knowledge only one study analyzed the specific relationship between knowledge of story structure and listening comprehension showing that this ability directly predicted listening narrative comprehension of very young children (three to four years old), controlling for receptive and expressive lexical competencies [60].

In conclusion, there is evidence that higher-level cognitive skills are directly and specifically related to listening narrative comprehension: being the understanding an integrative and constructive process, these broad cognitive skills guarantee coherence. Although their role has been examined and demonstrated extensively in school-age and as far as reading comprehension is concerned, further evidence is needed on the role of these skills in preschool narrative comprehension. Above all, it needs to be clarified whether already at this age their role is specific and direct in determining individual differences in listening narrative comprehension.

## 3. The Role of Linguistic Experience in Listening Narrative Comprehension

One of the innovative aspects of this work concerns the inclusion, within the multicomponent model of listening narrative comprehension, of the children’s linguistic experience. This has been studied in terms of variation in the amount of exposure to the majority language, which in this study is Italian. The variation in exposure is mainly because the children participating in the study come from both monolingual and multilingual contexts. Nowadays, most classes are composed of children who have a variable exposure to the majority language spoken at school. In this study, we decided to combine children who develop in monolingual and bilingual contexts since we were interested in examining whether variation in length of exposure to the majority language affects preschoolers’ performance in several cognitive and linguistic skills, related to performance in listening narrative comprehension task. We know that even a small variation in the length of exposure has the potential to give rise to differences in linguistic and cognitive skills [61].

Definition of bilingual exposure is more complex than a “yes/no” categorization and one of the sources of variation in populations exposed to more than one language is the amount of language exposure [62]. To date, there is no standard definition of bilingualism or a common standard for determining how to describe individuals in terms of this complex experience [63]. According to different authors [64], since bilingual children can differ from each other, the field should move away from monolithic bilingual vs. monolingual comparisons. This categorical distinction does not take into account individual differences in language experience [64]. Bilinguals may show individual differences in their linguistic competence in the majority language, based on age, the onset of language exposure, amount and quality of linguistic input, and circumstances under which language is acquired [61,65]. Luk and Bialystok stated that Language experience lies on a continuum: individuals are not categorically bilingual or monolingual [66]. According to this literature, instead of using a dichotomous classification in this paper, we used a continuous variable that takes into account children’s majority language experiences. Therefore, rather than defining children as monolinguals or bilinguals, we describe their exposure as monolingual or bilingual: the length of exposure (defined on a continuum) better explains individual differences in any cognitive and linguistic task than group classification (bi- vs. monolinguals). In the current work, we examined the role of the variation in the length of exposure to the majority language both in listening narrative comprehension and in the components related to narrative comprehension.

Few studies have directly assessed the role of the variation in the length of exposure to the community language in their acquisition of narrative skills and related components. Rodina [67], for instance, found that children growing up in a multilingual context with less exposure to the majority language (i.e., children who predominantly heard and spoke the native language) show low scores in narrative production (both in micro and macro structure) and production complexity if compared with children growing up in a monolingual context and children coming from predominantly majority language speaking homes. Other studies suggest that length of exposure to majority language is an important predictor of all microstructure measures, i.e., number of different words, sentence length, and grammaticality [9,68], whereas macrostructure measures (i.e., story structure or organization) evaluated with generation tasks are less influenced by the length of exposure. Narrative macrostructure tends to be relatively invariant across languages [7,69,70], suggesting that narratives of children growing up in multilingual contexts should display robust elements of story structure regardless of the language used for narration, and relatively insensitive to language level [8].

Length of exposure to the majority language could also play an indirect role in narrative comprehension, i.e., mediated by low and high-level cognitive and linguistic skills. Studies conducted so far indicated that, as far as lower-level linguistic skills are concerned, the length of exposure is a crucial factor in determining differences in language development. Children coming from multilingual families receive less exposure to each language than children coming from monolingual families because their parents need to divide language input between two languages [71]. Evidence suggests that the length of exposure to the majority language is linked to vocabulary, grammar development, and listening narrative comprehension [61,71,72,73]. The quantity of language exposure has a significant influence on the size of children’s vocabularies across the age range from 30 to 60 months, with the result that the children with multilingual exposure lag behind monolingual children in vocabulary [74]. Dicataldo and Roch found that variation in bilingual exposure (BE), both length and daily exposure to the host language, predicted specifically vocabulary and listening narrative comprehension in that language [61]. Variations observed among children are the result of differences in the linguistic structures that they hear and differences in the frequency of their exposure to these linguistic structures [75]. Moreover, it is known that the host language becomes increasingly important once children enter formal education in general and formal reading education in particular [76].

Concerning lower-level cognitive skills involved in listening comprehension, a large body of research has shown that bilingual exposure has a positive effect on cognitive control components that fall under the umbrella term executive functions (EFs) [61,77,78,79,80]. However, as Bialystok and colleagues pointed out [81], what is less clearly understood is exactly when and how these advantageous effects surface in the context of emerging bilingualism. Studies comparing children in immersive education programs with children in monolingual education have provided mixed results regarding the effect of length of exposure on EFs. Nicolay and Poncelet reported positive effects of a three-year immersion program on tasks assessing alerting, auditory selective attention, divided attention, and mental flexibility, thereby suggesting that three years of second-language immersion school experience produces some of the EF benefits associated with early bilingual exposure [82]. Purić, Vuksanović, and Chondrogianni [83], in their study, found that children with a higher amount of daily exposure to the second language, after only one year of exposure, obtain better scores on WM tasks. Thus, the nature of bilingual experience affects also the development of different EF components [84]. A possible interpretation of these findings could be that the level of language proficiency is the key variable through which language experience influences EFs’ development [83]. To the best of our knowledge, there are no data available on the relationship between linguistic experience and higher order cognitive skills involved in listening narrative comprehension.

## 4. The Current Study

This study aimed to examine, within the multicomponent model of comprehension, direct and indirect pathways of lower-order cognitive skills (working memory, attention, and inhibitory control), language skills (receptive vocabulary, syntactic knowledge, and rapid naming), higher-order cognitive skills (inferential abilities, theory of mind, and knowledge of story structure), and variation in the length of exposure to Italian, the majority language, in narrative comprehension evaluated with listening comprehension questions’ task.

This is the first study that has included such a complete set of components of listening narrative comprehension into a single study. Furthermore, for the first time, the model includes the role of the variation in the length of exposure to the language. Five alternative models of structural equations were examined and compared to empirically test plausible alternative paths systematically (as proposed by Kim in her work with children in Grade 1) to better specify how language and cognitive skills are directly and indirectly related to listening narrative comprehension [14]. With these models, all the possible relations between the three broad categories included in the multicomponent model of comprehension [1], namely, lower-order cognitive skills, lower-order language skills, and higher-order cognitive skills, and children’ performance in listening narrative comprehension task, have been tested including for the first time into the model also the role of the variation in the length of exposure to the majority language.

**Figure 1:**
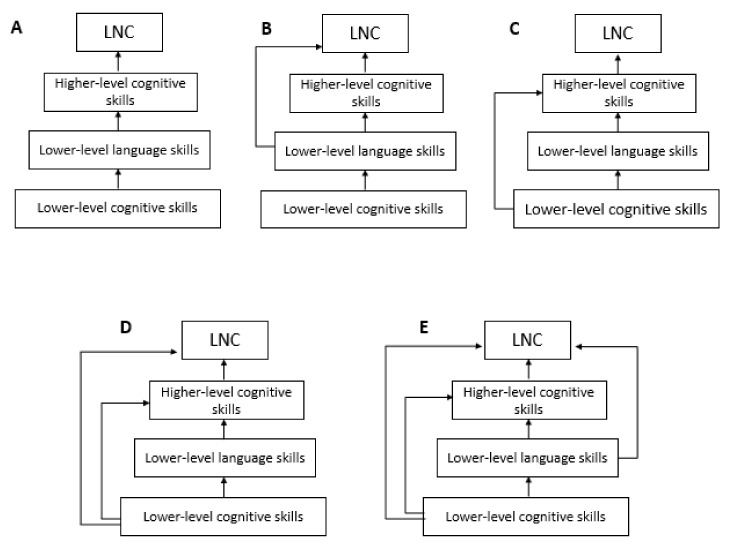
Five alternative models of hypothesized relations of cognitive and language skills to listening narrative comprehension. Panels (**A**–**E**) represent Models 1, 2, 3, 4, and 5, respectively. *Note:* LNC = Listening Narrative comprehension; higher-level cognitive skills = inferences, ToM and knowledge of story-structure; lower-level language skills = vocabulary, syntactic knowledge, and rapid naming; lower-level cognitive skills = WM, inhibitory control and attention shifting.

The logic in the construction of the predictive models of listening narrative comprehension considered both the presence of all the components combined in the three broad categories and, based on the existing literature, the evaluation of the type of role (direct or indirect) of each component on the listening narrative comprehension (Figure 1). As a result, both models testing complete mediation effects and models testing partial mediation effects were built following some previous studies that gave only piecemeal evidence. Last but not least, the mediated role of the variation in the length of exposure to the majority language on all the components of listening narrative comprehension is hypothesized.

### 4.1. Method

#### 4.1.1. Participants

This study has been approved by the Ethics Committee of the University of Padua–Department of Psychology, (protocol number 2064) on 1 December 2016. One hundred and fifteen children were recruited from 4 different schools in the metropolitan area of [removed for review], a medium-sized city in Northeast Italy. All children have received at least 2 years of formal language provided in educational settings. Parents were asked to sign a consent form if they agreed to take part and let their children take part. Parental permission forms were distributed to parents of all children of the age range 4–6 years in the selected schools. We handed out 150 consent forms and received consent for 115 children. From the original group, one child was excluded for hearing problems declared by parents and 3 for their incomplete language and cognitive assessment. The final group consists of 111 children (61 boys and 50 girls) aged between 44 and 75 months (mean = 5 years and 2 months, SD = 6.8 months).

All children of each class and their families were invited to take part in the study. Our group was therefore deliberately heterogeneous in terms of language or languages to capture the variability of children who are currently involved in the school system in the area in which the research was carried out and in particular to capitalize on the notion of language exposure as a continuous measure.

Background data, concerning SES and exposure to the majority language, namely, Italian, were collected through a questionnaire filled out by parents. We found that the group varied widely in socio-economic status measures. Years of education ranged from 5 to 22 for the mothers and from 5 to 20 for the fathers, with mean 13 years, equivalent to receiving a high school degree (*SD* = 3.3); household income ranged from € 18.000 to over € 41.000 with mean equal to € 30.340 (*SD* = € 8.317), which is equivalent to the Italian median family income (Istat, 2009).

The group varied also as far as the length of exposure to the majority language is concerned. Concerning the age of first exposure to Italian, we found that it was very wide: from 0 to 36 months (mean = 6.3; SD = 10.1 months). Starting from this information, we computed the length of exposure to the majority language, which indicates the precocity of potential bilingual exposure, which ranged between 24 and 75 months.

#### 4.1.2. Procedure

Children were tested individually in three sessions, 2 days apart, by 3 research assistants (undergraduate students with extensive experiences with children, including language and literacy assessments) in a quiet room of their school. All tasks were presented to children in Italian, in a fixed-order and each session lasted approximately 30 min. When a child showed signs of fatigue, the assessment was interrupted and resumed, from the breakpoint, in the following session. At the end of each session, children were thanked for their participation and prized with free play-time.

##### Listening Narrative Comprehension (Dependent Variable)

Listening Narrative comprehension was assessed through the standardized test TOR 3–8 for the Italian language [85]. This test evaluates listening narrative comprehension of children aged between 3 and 8 years of age. It consists of two short stories of equal difficulty and length, which are read individually to each participant. Listening narrative comprehension is evaluated using 10 questions per story, half of which are based on explicit information (textual questions) and half on information that requires inferences to be generated (inferential questions). The tester reads the stories and, to minimize the cognitive and memory load, he/she interrupts reading at two predetermined points and asks questions followed by a multiple-choice task (see Appendix A). The tester reads the answers and the children are asked to respond by choosing the correct answer out of four possibilities (pictures). Each correct answer is given one point. The final score consists of the sum of correct answers, 10 for each story, with a maximum score of 20. Raw scores are transformed into standard scores having mean = 10 and SD = 2. The internal reliability evaluated by calculating Cronbach’s alpha over items, reported in the manual, ranges from 0.52 to 0.72.

##### Lower-Order Cognitive Skills

Working memory: the backward digit span, WISC sub-test [86,87], was used to evaluate children working memory. A list of predetermined random numbers ranging from two to eight digits are read aloud. Backward digit span requires the simultaneous storage and processing of information in memory because the children had to repeat digit lists in reverse order. There are two trials for each digit length. The test begins with two numbers, increasing until the child fails at two consecutive digit sequences of the same length. The child’s digit span score is the total number of trials completed correctly (range 0–16), moreover, the longest list length correctly repeated in the two trials is defined as memory span (max 8). The reliability for the digit span task evaluated using the split-half procedure, as reported in the test manual, is 0.81.

Inhibitory control: Day and Night test, a subtest of FE-PS 2- 6 [88], was used to assess the ability to suppress a dominant response related to perceptual stimuli in the task while selecting and executing a competing, conflicting response. This task contains two decks of cards: the first contains 8 cards depicting a chessboard and 8 an X; the second deck contains 8 cards depicting a sun and 8 a moon. In the control condition, the tester trains the child to say “day” when there is an X card and “night” when there is a chessboard card. In the Inhibition condition (i.e., Stroop condition), the child has to say the word ‘day’ when viewing a card depicting a nighttime sky and to say ‘night’ when shown a picture of the daytime sky. Each child completes 16 trials for each condition that are scored 0 (incorrect) or 1 (correct). Three different scores are calculated: accuracy (range 0–16), speed (in seconds); inhibition score is given by subtracting the accuracy in the Inhibition condition from performance in the Control one (range −16 to 16). A smaller difference between the numbers of correct answers in the two phases corresponds to better performance. The reliability evaluated by calculating Cronbach’s alpha over the items, as reported in the tests’ manuals, is 0.85.

Attention shifting: Dimensional Change Card Sort (DCCS), a subtest of FE-PS 2- 6 [88], was used to assess children’s attention shifting. This task consists in sorting neutral cards based on characteristics of the object presented on cards. The DCCS consists of 3 decks of cards one for each phase and requires that children sort each card presented into one of two piles according to a rule provided by the experimenter. During the first phase (“shape game”), children are instructed to sort cards into the correct piles based on shape. In the second phase, children are told that the rule has changed and they now must sort cards based on color (“color game”). In the third phase (“border game”), children are told that cards with the border would be sorted according to the role of the “shape game”, while cards without the border would be sorted according to the role of the “color game”. The tester records how many cards the child can classify correctly in each trial: performances on the shape and color version are scored as the number of correct choices out of 6; performance on the border version is scored as the number correct choices out of 12. Total accuracy score was used in the analyses (range 0–24). The reliability, evaluated by calculating Cronbach’s alpha over the items, is 0.39. Although this value results low, it is comparable with values reported in a previous work, i.e., r = 0.36 (see [89]).

##### Lower-Order Oral Language Skills

Receptive vocabulary: The PPVT Revised is a standardized test, which evaluates receptive vocabulary [90]. It consists of a list of 175 words, but each participant is presented with a different number of words depending on his/her lexical knowledge. The word items are presented in increasing order of difficulty. Children are asked to point which out of four pictures best represents the target word pronounced by the tester. A basal level is defined based on the child’s ability to give 8 consecutive correct answers. Testing is then continued until the participant obtains 6 incorrect answers out of the last 8 words presented (ceiling level). Raw scores correspond to the number of correct answers. Standard scores are computed based on raw scores and chronological age: M = 100, SD = 15. The reliability evaluated using the split-half procedure, as reported in the test manual, is 0.88.

Syntactic knowledge: Prova di Valutazione della Comprensione Linguistica (PVCL; Test for the Evaluation of Linguistic Comprehension) is a standardized test for children aged between 3, 6, and 8 years which evaluates syntactic knowledge through sentence comprehension [91]. The test consists of a total of 78 illustrated sheets, each comprising 4 pictures, corresponding to 78 items divided into 6 protocols. Each protocol is adopted for a certain age range and contains between 12 and 16 sentences each. Sentences contained salient morphosyntactic cues, such as gender and number agreement, conjunction, negation, different types of phrasal structures (i.e., relative, passive, temporal). Children are required to choose which picture from among a set of four correctly represented the sentence spoken by the experimenter. Each sentence, taking into account the level of difficulty, at the time of test’s construction and standardization, has been given a specific score and the score ranges between 0 and 100. The score evaluates children’s overall performance taking into account the number of correct answers and the level of difficulty of each item. The reliability evaluated using the split-half procedure, is 0.75.

Rapid naming: Rapid naming subtest from the linguistic domain of *NEPSY-II* [92,93], was used to obtain measures of rapid semantic access and production of names of colors, shapes, and sizes. The child is shown a selection of colors and shapes; colors, shapes, and sizes; letters and numbers and has to name them in order as quickly as possible. Each correct or self-corrected answer is given one point. Accuracy (range 0–84), self-corrections (range 0–84), and speed (max 600 s) are recorded and used to calculate a combined score on a distribution with mean = 10 and SD = 3. The reliability evaluated using the split-half procedure, as reported in the test manual, is 0.81.

##### Higher-Order Cognitive Skills

Inferential abilities: An experimental task developed by Florit, Roch, and Levorato [15] was used to assess inferential abilities. The task consisted of ten items, each containing two short sentences read aloud referring to common and familiar events, followed by two inferential questions. Questions focused on two types of inferences: knowledge-based and text-based inferences. Knowledge-based inferences require information from previously acquired world-knowledge to be incorporated within the episode (e.g., “That day Piero could not wait to wear the swimsuit and play with scoop and bucket. Where he had gone that day?”), instead, text-based inferences are necessary to connect various pieces of information provided in the short episode and to identify their implicit relations (e.g., “Then Piero picked up the scoop and the bucket. He put the games in the bag; where are the scoop and the bucket?”). Answer to each question was evaluated on a 0–2-point scale: a complete incorrect answer was scored 0, whereas partially correct answer or answer provided after a clarification was scored 1, and fully correct answer was scored 2. Three scores were calculated: knowledge-based inferences score (range = 0–20), text-based inferences score (range = 0–20) and total inferences score (range = 0–40). The reliability, evaluated by calculating Cronbach’s alpha over the 10 items, was 0.65. This rather low alpha level is not surprising; the task consisted of different types of questions that aimed at evaluating the ability to generate two different types of inferences. Moreover, there were too few items of each type to obtain reliable assessments of homogeneity within each type.

Theory of Mind: False Belief task (adapted from [94]) was used to assess the capacity to attribute mental states to self and others. In this task, children are shown the “pasta box” and are asked to guess its content. Then, the tester shows the actual content of the box (i.e., pencils) and asks children to identify what the object is. In the control trial (“self” question) the tester asks “What did you think was there when you saw it?”. The second part of this task involves the ToM trial: another person came into the room for a while, took a look at the “pasta box”, and went out; after this, the tester asked the child what the other person thought would be in the box (“other” question). The score ranges between 0 and 2: either 1 (correct) or 0 (incorrect) is given to the “self” and “other” questions.

Knowledge of story structure: An experimental task was developed to evaluate the ability to infer complex semantic relationships between events. This task consists of six sets of pictured stories each composed of 6 pictures. The stories used are of increasing difficulty (for the number of details, events represented, etc.), so we started from the simplest set of pictures to the most complex ones, in a fixed sequential order. For each story, the children are asked to observe the pictures provided in a random order and to arrange them to create a story. In this task, children have to focus their attention on elements that may help them understand the pattern of the story and then identify, in the pictures, setting, characters, initiating event, reactions, attempts, and resolution, all elements that allowing to better understand stories.

Children are presented with an example set of 4 cards to practice with the task; if children are not able to order the example set, the experimenter shows them how to arrange the set explaining the meaning of the story depicted, to be sure they understood the task. Score ranges between 0 and 36: 1 point is assigned for each card arranged correctly and 0 for card arranged wrongly. Reliability, evaluated by calculating Cronbach’s alpha was 0.67.

##### Length of Exposure to Majority Language

As said, parents were asked to fill in a questionnaire to obtain information about the length of exposure to the majority language.

Parents were asked to indicate the age (in months) in which their child was exposed for the first time to Italian, i.e., when they started to talk in Italian with him/her (Age of Onset). Starting from this information we computed the “length of exposure” to the majority language as the difference between the age of onset to the majority language and age at the time of testing. Whereas for children growing in a monolingual context, this measure corresponds to their chronological age, for children growing in a bi or multilingual context may be not the same since some of children may have been exposed to the majority language later in time.

As said before, to date, there is no standard definition of bilingualism or a common standard for determining how to describe individuals in terms of this complex experience [63], thus rather than defining children as monolinguals or bilinguals using a dichotomous classification, we used a continuous variable that takes into account the variation of children’s majority language experiences [61].

### 4.2. Data Analysis Strategy

Structural equation modeling was primary data analysis strategy. Each ability was assessed by a single measure and therefore observed variables were used for defining each skill. The hypotheses have been tested by path analysis modeling, which is an adequate and powerful approach for examining direct and mediated relations between observed variables. This approach allows modeling multiple predictors and multiple outcomes, which in turn can become predictors in a single model [95]. Model fits were evaluated by using the following multiple indices: chi-square statistics, comparative fit index (CFI), Nonnormed Fit Index (NNFI), Akaike’s information criterion (AIC), Bayesian information criterion (BIC), root mean square error of approximation (RMSEA), and standardized root mean square residuals (SRMR). Typically, RMSEA values below 0.08, CFI and NNFI values equal to or greater than 0.95, and SRMR equal to or less than 0.10 indicate an acceptable model fit. RMSEA values between 0.05 and 0.08, CFI and NNFI values equal to or greater than 0.97, and SRMR equal to or less than 0.05 indicate a good model fit [96]. Path analysis were conducted with R package lavaan, version 0.4–11 [97].

### 4.3. Models’ Description

The first model tested was a complete mediation model in which lower-order cognitive skills were hypothesized to be directly related to lower-order language skills, which in turn, were directly related to higher-order cognitive skills; the latter were hypothesized to be directly related to narrative comprehension. To describe a final comprehensive model that includes direct and indirect pathways to listening narrative comprehension, the other models differed in terms of how lower-order cognitive and language skills were specified to have direct relations to higher-order cognitive skills and to listening narrative comprehension.

Model 2 was the same as Model 1 but here lower-order language skills were hypothesized to have also direct relations to listening narrative comprehension over and above higher-order cognitive skills. Previous studies have shown that vocabulary and grammar knowledge were directly related to narrative listening comprehension over and above ToM [47].

Model 3 was the same as Model 1 but here lower-order cognitive skills were hypothesized to have also direct relations to higher-order cognitive skills over and above lower-order language skills. Previous studies have shown, for instance, that to make an inference, information from the text and previous knowledge had to be recalled and integrated thus limited lower-order cognitive skills my produce inference difficulty which in turn will constrain narrative comprehension [98]. In Model 4, according to the evidence that working memory makes a unique contribution to listening narrative comprehension after controlling for verbal ability assessed through vocabulary tasks [16], lower-order cognitive skills were hypothesized to be directly related to higher-order cognitive skills and narrative listening comprehension.

Finally, in Model 5 all direct and indirect relations were allowed from lower-order language and cognitive skills to higher-order cognitive skills and listening narrative comprehension. The comparison of this model to all the previous allows drawing the complex picture of direct and indirect relations between component skills and listening narrative comprehension.

Based on previous findings of studies that have adopted a multicomponent approach [14,15,16,33,47], we predicted that the Model in which all direct and indirect relations were allowed from lower-order language and cognitive skills to higher-order cognitive skills and listening narrative comprehension, thus Model 5, would have the best fit with our data. However, since this is the first time that such a set of language and cognitive skills are analyzed in a comprehensive model, we do not have specific predictions about relation across skills.

Finally, concerning the effects of the variation in the length of exposure to the majority language, we predicted that the length of exposure to the majority language would account for individual differences in various skills. Since this is the first time that such a prediction is tested, we do not put forward specific predictions; therefore, we tested all the possible relations to component skills and to listening narrative comprehension. Model 1 were included all possible relations between the variation in the length of exposure to the majority language to lower-level language and cognitive skills, to higher-level cognitive skills, and listening narrative comprehension. In the subsequent four models, only the significant relations resulted from Model 1 were included to test more parsimonious models in which these nonsignificant paths of between lengths of exposure to the majority language were removed.

## 5. Results

### 5.1. Descriptive Statistics and Preliminary Analyses

Table 1 shows minimum, maximum, means, standard deviations, Skewness, and Kurtosis of all the variables considered in the current work. In Table 1 raw scores are reported and, where available, standardized scores are taken from test manual to facilitate the interpretation of the mean performance. Finally, for performance in the ToM task, are reported raw scores transformed in z-scores.

The group showed a low average score in the standardized vocabulary task (PPVT-R): the average performance lay under the lower boundary of the range appropriate for age [90] while the standard deviation was comparable to that of the national standardization sample [15]. Performance on the majority of other tasks covered a large range of scores and none suffers from ceiling effects. On the other hand, we found a floor effect on the Digit span task.

The distributions of the majority of the variables approached symmetric, with exception of DCCS task whose values of skewness and kurtosis were high. Inspection of frequencies of scores indicated that large kurtosis value was because 75% of children scored between 16 and 21. Given that z-score transformations did not change the overall shape of the DCCS’ score distribution, and that the spread of scores was across a band and not on a single score, DCCS’s raw scores were used. In the further data analyses, for the majority of variables, raw scores were used whereas z-scores for ToM task and a combined score for rapid naming was used.

Zero-order correlations among measures are presented in Table 2. For the sake of clarity and for the purposes of the current work, the correlations between each group of independent variables on one hand and listening narrative comprehension, on the other hand, are commented on. All the other relations can be observed in Table 2. For what concerns lower-order cognitive skills, working memory and inhibitory control were weakly related to listening narrative comprehension (respectively *r* = 0.27 and *r* = 0.26), whereas attention was not significantly related to listening narrative comprehension. As far as lower-order language skills, moderately interrelated (0.26 < *r* < 0.50), vocabulary has the highest correlation with listening narrative comprehension (*r* = 0.68), while syntactic knowledge and rapid naming were moderately related with listening narrative comprehension, respectively *r* = 0.46 and *r* = 0.41.

With regards to the correlations between higher-order cognitive skills, inferential ability and knowledge of story structure were moderately correlated with listening narrative comprehension (respectively *r* = 065; 0.53), whereas Theory of mind shows a weak but significant correlation with listening narrative comprehension’ score (*r* = 0.33). Magnitudes of correlations across different skills are similar to results of previous studies conducted in Italy and other countries [3,43,47].

Finally, we found a moderate correlation (*r* = 0.42) between listening narrative comprehension and length of exposure to majority language whereas the correlations with the other cognitive and linguistic skills ranged between 0.19 and 0.53 and are all significant (see Table 2).

### 5.2. Direct and Indirect Pathways of Language Exposure to the Majority Language, Cognitive and Language Skills to Listening Narrative Comprehension

This work aims to examine, within a multicomponent model, direct and indirect pathways of a large and comprehensive set of linguistic and cognitive skills involved in listening narrative comprehension in children, aged between 44 and 75 months, growing in monolingual and multilingual contexts and thus with a different length of exposure to the majority language.

First, we tested direct relations between length of exposure to majority language, lower-order cognitive and language skills, higher-order cognitive skills, and listening narrative comprehension. Length of exposure was found to be directly related to all lower-level cognitive skills, receptive vocabulary, and knowledge of story structure thus in all the models tested we included only these significant relations whereas non-significant relations were not included.

For our aim, five alternative nested path-models of hypothesized relations between lower-order cognitive skills, lower-order language skills, and higher-order cognitive skills to children’s performance in listening narrative comprehension were fitted and compared. Additionally, we tested direct relations between length of exposure to, lower-order cognitive and language skills, higher-order cognitive skills, and listening narrative comprehension. Table 3 shows Models fit information.

Model 1, the complete mediation model, Model 3 in which lower-order cognitive skills were hypothesized to have direct relations to higher-order cognitive skills over and above lower-order language skills, and Model 4 in which lower-order cognitive skills were hypothesized to be directly related to higher-order cognitive skills and listening narrative comprehension, did not fit the data very well.

Model 2 in which lower-order language skills were hypothesized to have direct relations to listening narrative comprehension over and above higher-order cognitive skills has shown an acceptable fit to the data (χ^2^ (22) = 27.91, *p* = 0.17; CFI = 0.97; AIC = 6188; BIC = 6305; RMSEA = 0.05; SRMR = 0.058).

However, Model 5 in which all direct and indirect relations were allowed from lower-order cognitive and language skills to higher-order cognitive skills and narrative listening comprehension, shown a good fit to the data (χ^2^ (10) = 11.44, *p* = 0.32; CFI = 0.99; AIC = 6196; BIC = 6345; RMSEA = 0.03; SRMR = 0.045).

**Figure 2:**
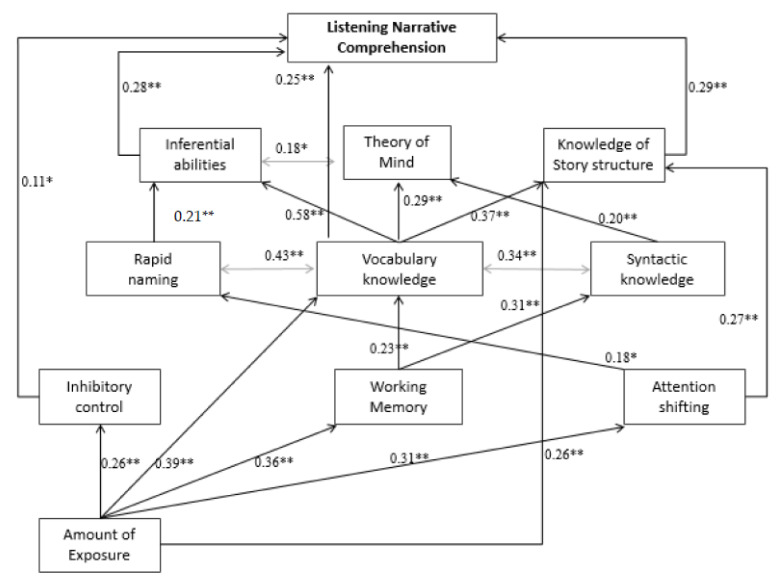
Final model of relations of length of exposure to the majority language, lower-order cognitive skills, lower-order language skills, and higher-order cognitive skills to listening narrative comprehension. Only statistically significant paths (solid lines) and statistically significant covariance (gray lines) are reported. Note: * *p* < 0.05; ** *p* < 0.01.

The chi-square difference test between models supports that Model 5 is superior if compared with Model 1, Model 3, and Model 4, whereas there is no difference between Model 5 and Model 2. As reported below, Model 2 showed an acceptable fit to the data with lower AIC and BIC values if compared with Model 5 because these values penalize a high number of estimated parameters rewarding parsimony. However, CFI and NNFI values and in particular RMSEA and SRMR values, i.e., measures of overall model fit that indicate to which extent a structural equation model corresponds to the empirical data, supports that Model 5 is the superior model even if compared with Model 2. For all these reasons, Model 5 was chosen as the final model. Completely standardized path coefficients of Model 5 are shown in Figure 2 [97].

The total amount of variance in listening narrative comprehension explained by our comprehensive multicomponent model was 0.60. The unique and independent variance explained by each component skills included in the model was as follows: lower-order cognitive skills: 0.10 attention shifting, 0.07 inhibitory control and 0.13 working memory; lower-order language skills: 0.07 rapid naming, 0.31 receptive vocabulary and, 0.17 syntactic knowledge; higher-order cognitive skills: 0.37 knowledge of story structure, 0.52 inferential abilities and 0.25 in Theory of Mind. What follows is the description of the direct and mediated relations between the component skills and listening narrative comprehension.

The length of exposure to the majority language did not directly predict listening narrative comprehension, after the other cognitive and linguistic component skills were controlled for. Variation of the length of exposure produces differences in performance on tasks evaluating lower-level cognitive and language skills, namely, inhibitory control, WM, attention shifting, and receptive vocabulary. Additionally, it is directly related to knowledge of story structure, a higher-order cognitive skill. These paths fully mediated the relation between the length of exposure to the majority language and listening narrative comprehension in that language. In detail, the indirect path between the length of exposure and listening narrative comprehension is mediated by its direct relation to all the lower-order cognitive skills (0.26 < *β* < 0.36, *p* < 0.01), receptive vocabulary (*β* =.36, *p* < 0.001, 95% CI [0.02, 0.06]), and knowledge of story structure (*β* = 0.26, *p* < 0.05, 95% CI [0.01, 0.05]).

Concerning lower-order cognitive skills we found a direct, even though weak, path between inhibitory control and listening narrative comprehension (*β* = 0.11, *p* < 0.05, 95% CI [0.02, 0.23]). The other lower-order cognitive skills were related indirectly to listening narrative comprehension: in detail, attention shifting was related through knowledge of story structure (*β* = 0.21, *p* < 0.01, 95% CI [0.03, 0.37]) while working memory through the receptive vocabulary (*β* = 0.24, *p* < 0.01, 95% CI [0.07, 0.39]).

As far as lower-order language skills are concerned, results show an only direct path between receptive vocabulary and listening narrative comprehension (*β* = 0.25, *p* < 0.01, 95% CI [0.06, 0.45]). The other paths, namely, between rapid naming and syntactic knowledge on one hand and listening narrative comprehension, on the other, were fully mediated by higher-order cognitive skills or not significant. In detail, rapid naming was related to listening narrative comprehension through inferential skills (*β* = 0.21, *p* < 0.01, 95% CI [0.07, 0.36]), whereas was found neither direct nor indirect path between syntactic knowledge and listening narrative comprehension. It is worth noting that receptive vocabulary was also related to listening narrative comprehension through indirect paths. Higher-order cognitive skills, namely, inferential abilities (*β* = 0.57, *p* < 0.001, 95% CI [0.41, 0.75]) and knowledge of story structure (*β* = 0.24, *p* < 0.05, 95% CI [0.03, 0.46]), in fact, mediated the effects that vocabulary has on listening comprehension.

Finally, concerning higher-order cognitive skills, results show that inferential abilities and knowledge of story structure, were both directly and independently related-respectively *β* = 0.28, *p* < 0.001, 95% CI [0.11, 0.45] and *β* = 0.30, *p* < 0.001, 95% CI [0.15, 0.44] to listening narrative comprehension, after all other predictors were accounted for.

For the sake of clarity, although not within the aim of this study, we report also significant paths among all the components evaluated in this model. In detail: attention shifting was directly related to knowledge of story structure (*β* = 0.21, *p* < 0.01, 95% CI [0.03, 0.37]) and rapid naming(*β* = 0.20, *p* < 0.05, 95% CI [0.02, 0.38]); working memory was directly related to performance in tasks aimed to assess lower-order language skills, namely, receptive vocabulary task (*β* = 0.24, *p* < 0.01, 95% CI [0.07, 0.39]) and syntax knowledge task (*β* = 0.31, *p* < 0.001, 95% CI [0.13, 0.48]). As far as lower-order oral language skills, performance in receptive vocabulary task was related to performance in all tasks aimed to assess higher-order cognitive skills, namely, inferential abilities task (*β* = 0.57, *p* < 0.001, 95% CI [0.41, 0.75]), theory of mind task (*β* = 0.30, *p* < 0.01, 95% CI [0.09, 0.51]), and knowledge of story structure task (*β* = 0.24, *p* < 0.05, 95% CI [0.03, 0.46]); rapid naming was related only to inferential abilities (*β* = 0.21, *p* < 0.01, 95% CI [0.07, 0.36]), whereas syntactical knowledge task was directly related to Theory of Mind (*β* = 0.20, *p* < 0.05, 95% CI [0.02, 0.39]).

## 6. Discussion

The current study was designed to examine, within the multicomponent model of comprehension, direct and indirect pathways of lower-order cognitive skills, language skills, higher-order cognitive skills, and variation of the exposure to the majority language, in narrative comprehension evaluated with listening comprehension. This is the first study that has included such a complete set of components of listening narrative comprehension into a single study. Moreover, for the first time, the model included the role of the length of exposure to the majority language.

Five path models were tested. The best-fitting model resulted in the one which allowed all the direct and indirect paths from all variables included in the model. Results indicated that the components included in the model explained a total of 60% variance in listening narrative comprehension in Italian, in children aged between four and six years and that the relations among all these skills reveal an extremely intricate picture.

The main innovation of this study is twofold: first, this is the first study in which such a large number of components of listening narrative comprehension are included; second, to our knowledge, this is the first work that considers, alongside the cognitive and linguistic components, the role of linguistic experience with the majority language, in listening narrative comprehension. In our view, this allows defining a comprehensive and complete multicomponent model of listening narrative comprehension in preschool children exposed, in a different amount, to the language of context.

These two main contributions are discussed in the following paragraphs in relation to existing literature and their innovative contribution.

### 6.1. The Comprehensive Multicomponent Model of Listening Narrative Comprehension in Preschool Age

As stated before, except for syntactic knowledge and ToM, all the components included in the model are relevant for listening narrative comprehension in the age range considered (four to six years) and predict listening narrative comprehension in the majority language, through direct or mediated paths.

It has been shown that direct influence comes from all three broad categories of components: lower-level cognitive and linguistic skills and higher-level cognitive skills. The direct role of inhibition in listening narrative comprehension suggested that the ability to inhibit attention to irrelevant details is essential for integrating information in the context of meaningful stories. This result is consistent with a recent study underlining that inhibitory control represents a necessary cognitive skill to build a coherent global representation of text meaning [23]. In this study, we expanded the result to participants that differ for the length of exposure to the majority language.

Among language skills, vocabulary has both direct and mediated roles in determining individual differences in listening narrative comprehension. As reported in numerous previous studies, vocabulary appears as a core language ability for successful listening narrative comprehension [3,41,42]. Additionally, receptive vocabulary produced important differences in high-level cognitive abilities, which in turn predict listening narrative comprehension. This is consistent with previous studies: vocabulary knowledge allows to arrange more concepts and generate inferences and fosters the knowledge of story structure [14,33,99,100]. Interestingly, although the direct and mediated role of receptive vocabulary in listening narrative comprehension has not been surprising, we moved forward by showing that this role is crucial even after considering the individual differences in linguistic exposure to the majority language.

The current findings emphasize the importance of higher-order cognitive skills for successful listening narrative comprehension in preschool children having different exposure to the majority language. We found that both inferential abilities and knowledge of story structure were related, independently and directly, to listening narrative comprehension. Inferential abilities are important for listening narrative comprehension since they allow building a local and global coherence [41,54,101], but not all studies have found a direct influence of inferential skills in listening comprehension. In fact, some studies found that the role of inferential skills in listening comprehension during preschool age is mediated by language skills [33]. Some authors have suggested that only by the end of preschool age and during the transition to primary school does the role of inferential skills become unique and specific, beyond the role of language skills [41]. The relevant contribution of the present work is the evidence that inferential abilities, despite the low internal consistency of our inference task, resulted to play a direct role in listening narrative comprehension over and above not only language skills, but also language exposure to the majority language. The contribution that inferential abilities have in listening comprehension, could be probably greater than observed here. Future studies will need to further investigate this finding, through longitudinal studies to better clarify the potential causal role played by integrative skills, in successful narrative comprehension.

Within the broad category of higher-level cognitive skills, alongside inferential abilities, knowledge of story structure was also directly related to listening narrative comprehension showing that children’s ability to arrange correctly a story, contributed to better listening narrative comprehension. Knowledge of story structure acts to organize the text, make predictions, support inference generation, and construction of a coherent mental representation of the text [102]. The role of knowledge of the story structure has not been extensively studied. An exception is made by the study conducted by Zampini and colleagues [60] in which a relationship between knowledge of story structure and listening comprehension was found only in very young children (three to four years old) but not in five-year-old children. Future studies should clarify the existence of a direct role of this skill in narrative comprehension. If this will be confirmed, fostering the knowledge of story structure could become a target skill in interventions aimed at improving narrative comprehension skills.

In summary, this work has highlighted the direct contribution of various components taking into account the differences related to language exposure. Alongside these direct paths, the other components affect the listening narrative comprehension through indirect pathways. Among the lower-level cognitive abilities, both attention control and working memory predict listening narrative comprehension, the former through the knowledge of story structure and the latter through receptive vocabulary. Both these paths highlight the cascading effect of basic attentional and memory resources on language and higher-order cognitive skills which in turn affect successful narrative comprehension. Concerning working memory, our findings are consistent with a recent study that showed that the relationship between working memory and listening comprehension was mediated by other linguistic and cognitive skills but discrepant from other studies that found that working memory was directly related to listening narrative comprehension [16,47]. Speculatively, the current results, which have to be replicated in future works, may be interpreted by the fact that the participants of the current study have had a variable length of exposure to Italian that was a native language for some but not for all participants. This variation could have had an important impact on the way in which cognitive skills predict the other components. Regarding attentional control, we did not find other works that included this component in the model of listening comprehension, and the current finding will have to be replicated with further data. However, it seems reasonable to speculate that attentional control is an important cognitive resource for an adequate performance of the story structure task which requires focusing attention on relevant elements and relations to order a story.

Finally, an indirect path, which has not yet been established, emerged between rapid naming and listening narrative comprehension, through inference abilities. In a previous longitudinal study, Parilla and colleagues [49] found that rapid naming in kindergarten directly predicted reading comprehension in the first and third grade. Rapid naming reflects a global developmental change in the speed with which many cognitive processes are executed. According to this view, the correlation between rapid naming and reading reflects the fact that both are linked to age-related changes in processing speed (Kail et al., 1999). As children develop during preschool and school years, they process information more rapidly [103], thus this developmental change contributes also to inferential ability in terms of speed of inference generation and increasing accuracy [104]. The contribution of the current work is that for the first time, the role of rapid naming was extended to preschool children and to listening narrative comprehension.

Although the non-significant results should not be commented on, we consider it relevant to spend few words to speculatively comment on the possible reasons for the lack of direct and indirect relationships between syntactic knowledge and TOM on the one hand and listening narrative comprehension on the other. These two components have been included in the multicomponent model, as the existing literature, at least some studies, have highlighted their contribution to narrative comprehension. It should be noted that, nowadays, there is no agreement on their effective contribution to narrative comprehension: some studies show their contribution [105,106], while others do not show a significant contribution [15]. The present results are in line with these latter studies. Finally, it is relevant to point out that, although syntactic knowledge and TOM do not appear to have direct or mediated effects with narrative comprehension, they are both relevant perhaps, for the other components included in the model, which in turn have a direct or mediated relationship with narrative comprehension. This calls for the need to obtain further empirical data in this regard.

### 6.2. The Role of Linguistic Exposure in Listening Narrative Comprehension

The second relevant and innovative contribution of our study concerns the fact that for the first time the role of language experience, measured in terms of length of exposure to the majority language, has been considered in a multicomponent model of listening narrative comprehension. The study participants included classes of children from both monolingual and multi-lingual backgrounds and, rather than treating language exposure as a dichotomous variable that distinguishes between bilinguals and monolinguals, we used a continuous variable since participants differed in terms of the length of exposure to the majority language, which for some corresponded to chronological age, but for others was very variable. Some previous studies have already used similar methodologies in trying to define the complexity of language exposure [61,62]. We took a step further in examining the role of variation in the length of exposure to the majority language conceived in this way in a multicomponent model of listening narrative comprehension. The variability in the amount of exposure to the majority language does not appear to have a direct effect on the outcome variable, namely, listening narrative comprehension, once that all the other components were accounted for. However, as expected, the differences in the length of exposure to the majority language have effects on all three broad categories of components of listening comprehension. Indeed, length of exposure predicts lower-level cognitive skills, namely, inhibition control, attention shifting, and working memory. As discussed previously, cognitive resources, in turn, guarantee adequate narrative comprehension. The role of the length of exposure to majority language in cognitive skills is not a new finding and reflects what has been found in previous works [82,83]. However, the contribution of this work is to add a new piece of knowledge: the effect that early language experience has on attentive and working memory resources has a cascading effect on narrative understanding. This should be taken into account especially in the case of children who come from multilingual backgrounds and show poor narrative comprehension skills.

The role of language exposure does not stop here. In fact, the results suggest that the length of exposure predicts receptive vocabulary. Again, this is not a new result: the literature agrees in demonstrating consistently that the development of vocabulary is affected by language exposure [71,72,74]. As we discussed earlier, vocabulary is confirmed to be the core linguistic skill in listening narrative comprehension, and considering that exposure to the majority language predicts the vocabulary size, it is relevant to keep in mind that narrative comprehension could also be influenced by variation in the length of exposure to the majority language.

A surprising result that emerged in the current work is that, contrary to what we expected, the length of exposure to the majority language predicts not only low-level linguistic and cognitive skills but also the knowledge of story structure, which is considered a high-level cognitive skill. To our knowledge, there are no studies that have analysed the relationship between language exposure and knowledge of story structure, and for this reason, it is necessary to have further empirical evidence in favor. What we offer here is an attempt to interpret, purely speculatively this result. It may be that the amount of exposure facilitates the construction of knowledge about story structure, as exposure to language also presupposes exposure to a greater number of narratives in that language. In any case, since knowledge of the structure of the story directly accounts for individual differences in listening narrative comprehension, it is relevant to take into account that the variation in the experience with the majority language could mediate this relationship.

Taken together, the current findings suggest that length of exposure is essential for the lower-order cognitive and language skills, which in turn are essential for higher-order cognitive skills with a cascading effect on listening narrative comprehension and therefore should be included as a component of listening narrative comprehension.

## 7. Conclusions

Listening narrative comprehension is a complex linguistic and cognitive skill, and according to recent findings and theoretical models, it is considered a multicomponent ability that involves numerous language and cognitive components [1,3,15]. Listening narrative comprehension develops in a gradual process, which starts in early childhood and improves with the development of each component involved as well as individual experiences [3]. Several studies investigated the direct and indirect effects of each component in children’s listening narrative comprehension before school age, providing piecemeal evidence about structural relations among listening narrative comprehension’s components. However, structural relations of cognitive and language skills with listening narrative comprehension in young children, before formal reading, are still not clearly defined as well as the effect of language experience on narrative comprehension and its components have so far never been studied [23]. The present study is unique in examining such a large number of cognitive and language components on listening narrative comprehension, keeping into account individual differences in length of exposure to the majority language. Moreover, it provides new evidence about the interaction between each of the components involved and the impact of all the components, to give rise to individual differences in young children’s performance in listening narrative comprehension.

Understanding these structural relations among abilities involved in listening narrative comprehension, before formal school education, is important from a theoretical and practical point of view. From a theoretical point of view to gain insight relative to paths of relations (direct and mediated) of language and cognitive skills and language experience involved in listening comprehension. From a practical point of view, implications may direct educational efforts to increase pre-readers listening comprehension, taking into account language and cognitive skills, also environmental factors that produce individual differences in children’s performance.

Few limitations and related future directions must be worth noting. First, because the data were obtained at one point in time, it is impossible to draw firm conclusions about the development of listening narrative comprehension in young children. The current findings are concurrent and therefore causal inferences cannot be drawn. The directions of relations in the present study were based on theoretical models and previous findings from studies using a multicomponent approach; therefore, further longitudinal studies, as well as experimental studies, are needed to determine directional and causal nature of relations among components of listening narrative comprehension examined in the present study. Furthermore, since narrative comprehension ability develops in a gradual process with the development of lower and higher-order components and the contribution of each component, as well as the relations between these skills gradually changes [107], developmental level of children is another aspect to examine in future studies.

Second, due to the time and resource constraints, single tasks were used to assess language, cognitive skills, and listening narrative comprehension thus observed variables were used for each skill. In future studies, it would be ideal to assess language, cognitive skills, and listening comprehension with multiple tasks creating latent variables to reduce the measurement error and to obtain stronger results.

Third, the number of participants, since the conceptual model includes 45 parameters, and according to Kline [108], the number of participants to a parameter should be 10:1 and should not fall below 5:1. The results of the present study are preliminary and should be taken with caution. Further studies with more participants, due to the complexity of the model tested, are needed to determine if these exploratory results are stable and robust. This is particularly important with regard to future analysis of the effects of exposure to the majority language: the group of participants in this study shows variability in the length of exposure; however, there is a notable misalignment, as the majority of participants come from predominantly monolingual backgrounds. Anyway, since even with reduced variability in the amount of multilingual exposure, relevant results have emerged, it is recommended to replicate these results with a group of participants with greater variability in the language exposure experience. Additionally, since we recognized that our measure of language exposure is quite raw, future studies should add more parameters to estimate the quantity of exposure to each language, such as the quantity of daily input and the quality of the input in both languages.

An additional issue concerns the fact that the assessment of language and cognitive skills was carried out with tasks in the language of the context. Clearly, children less exposed to the majority language are disadvantaged in performance on tasks in their non-dominant language and thus their performance, not only on linguistic skills but also on cognitive skills may have been influenced by the type of task used.

Further studies are needed to explore this aspect and should assess at least cognitive skills with non-verbal tasks to mitigate this issue. Despite these limitations, the current results provided an important contribution in the description of a comprehensive multicomponent model of listening narrative comprehension, which includes linguistic cognitive and experiential components considering both direct and mediated relations among these skills. Practically, the present study contributes to a more specific understanding of which skills are relevant to listening comprehension at this early age and therefore should be targeted by early intervention to increase pre-readers listening comprehension. Narrative comprehension is an important ability related to school readiness and, being the most important factor in predicting later reading comprehension [109,110], strongly related to later academic success.

## Figures and Tables

**Table 1:** Descriptive statistics and Characteristics of the participants.

Variable	Min	Max	Mean	sd	Skewness	Kurtosis
Age (in months)	44	75	62	6.8	−0.05	−0.78
Length of exposure to Italian (in months) Dependent variable	24	75	60	8.8	−1.4	5.2
TOR 3–8 standard score (M = 10; sd = 2)	7	15	10.5	1.8	−0.10	−0.73
TOR 3–8 raw score (range 0–20)	3	19	11.5	4.1	−0.27	−0.99
Lower-order cognitive skills						
Backward Digit span (range 0–8)	0	4	1.45	1.14	−0.17	−1.18
Day & Night (range −16–16)	−11	14	1.8	4.4	0.91	1.9
DCCS (range 0–24)	7	24	18.4	2.9	−1.01	3.8
Lower-order oral language skills						
PPVT-R standard score (M = 100; sd = 15)	53	118	82.6	13.4	0.32	−0.34
PPVT-R raw score (range 0–175)	6	109	60.3	23.2	−0.27	−0.04
PVCL standard score (range 0–100)	11	93	55	19.2	0.13	−0.73
Rapid -naming combined score (M = 10; sd = 3)	4	16	9.69	2.8	−0.18	−0.56
Higher-order cognitive skills						
Inferential abilities (range 0–40)	1	31	15.5	7.36	−0.13	−0.63
Theory of Mind (z score)	−1.7	1.1	0	.99	−0.30	−0.81
Knowledge of story structure (range 0–36)	2	30	14.8	7.06	0.31	−0.90

**Table 2:** Correlations among measures.

	1.	2.	3.	4.	5.	6.	7.	8.	9.	10.	11.
1. Listening narrative comprehension (Tor 3–8)	-	0.27 **	0.26 **	0.15	0.68 **	0.46 **	0.41 **	0.65 **	0.33 **	0.53 **	0.42 **
2. Working memory (Backward Digit span)		-	−0.19 *	0.26 **	0.39 **	0.38 **	0.22 *	0.24 *	0.24 **	0.38 *	0.36 **
3. Inhibitory control (Day & Night)			-	0.01	−0.27 **	−0.07	−0.09	−0.15	−0.01	−0.13	−0.26 **
4. Attention (DCCS)				-	0.20 *	0.27 **	0.26 **	0.19 *	0.11	0.37 **	0.30 **
5. Receptive Vocabulary (PPVT-R)				-		0.50 **	0.48 **	0.71 **	0.45 **	0.45 **	0.53 **
6. Syntactic knowledge (PVCL)						-	0.26 **	0.40 **	0.39 **	0.36 **	0.25 **
7. Rapid naming							-	0.49 **	0.30 **	0.13	0.20 *
8. Inferential Abilities								-	0.45 **	0.34 **	0.39 **
9. Theory of Mind									-	0.19 *	0.19 *
10. Knowledge of Story structure										-	0.49 **
11. Length of exposure											-

Note: * *p* < 0.05; ** *p* < 0.01.

**Table 3:** Model fit comparisons.

Model	χ^2^ (df), p	AIC	BIC	NNFI	CFI	RMSEA	SRMR	Δχ^2^ (Δdf)	ΔAIC
1	43.66 (25), <0.01	6198	6307	0.89	0.94	0.082	0.066		
2	27. 91 (22), 0.17	6188	6305	0.95	0.97	0.052	0.058	M1 vs. M2 = 15.7 (3), *p* < 0.001	9.74
3	31.63 (16), <0.05	6204	6337	0.86	0.96	0.094	0.059	M2 vs. M3 = −3.72 (6), *p* = 1	−15.7
4	26.64 (13), <0.05	6205	6346	0.85	0.96	0.097	0.055	M3 vs. M4 = 4.98 (3), *p* = 0.17	−1.02
5	11.44 (10), 0.32	6196	6345	0.98	0.99	0.036	0.045	M1 vs. M5 = 32.2 (15), *p* < 0.001	2.21
								M2 vs. M5 = 16.4 (12), *p* = 0.17	−7.53
								M3 vs. M5 = 20.2 (6), *p* < 0.001	8.18
								M4 vs. M5 = 15.1 (3), *p* < 0.001	9.19

## Data Availability

The datasets used during the current study are available from the corresponding author on reasonable request.

## Appendix A. Story and Example of Questions in Narrative Listening Comprehension Task, (TOR 3–8)

Once upon a time, there was a monster who lived in a cave. The monster was covered in fur: on his arms, on his body, everywhere. He was so lazy that he only ate the flowers that were near him. Therefore, he became very weak and could hardly walk.

(T)1. What did the monster eat?

** (1) Mushrooms

(2) Flowers *

(3) Potatoes

(4) Leaves

(I)2. Why was he weak?

(1) Because he was covered in fur

(2) Because he was bad

(3) Because he ate only flowers *

(4) Because he lived in a cave

*Legend: (T) = Textual questions; (I) = Inferential questions;*

* = correct answers.

** the answers were accompanied by pictures.
